# Molecular Effects of Elongation Factor Ts and Trigger Factor on the Unfolding and Aggregation of Elongation Factor Tu Induced by the Prokaryotic Molecular Chaperone Hsp33

**DOI:** 10.3390/biology10111171

**Published:** 2021-11-12

**Authors:** Minho Keum, Dai Ito, Mi-Seong Kim, Yuxi Lin, Kyeong-Hyeon Yoon, Jihoon Kim, Sung-Hee Lee, Ji-Hun Kim, Wookyung Yu, Young-Ho Lee, Hyung-Sik Won

**Affiliations:** 1 Department of Biotechnology, Research Institute (RIBHS) and College of Biomedical and Health Science, Konkuk University, Chungju 27478, Korea; alsgh2747@kku.ac.kr (M.K.); 93mitong@naver.com (M.-S.K.); rudgus5392@naver.com (K.-H.Y.); jihoon0821@naver.com (J.K.); 2 BK21 Project Team, Department of Applied Life Science, Graduate School, Konkuk University, Chungju 27478, Korea; 3 Department of Brain and Cognitive Sciences, DGIST, Daegu 42988, Korea; dai.ito.osaka@gmail.com (D.I.); wkyu@dgist.ac.kr (W.Y.); 4 Research Center of Bioconvergence Analysis, Korea Basic Science Institute, Ochang, Cheongju 28119, Korea; linyuxi@kbsi.re.kr; 5 College of Pharmacy, Chungbuk National University, Cheongju 28160, Korea; suzukaze@naver.com (S.-H.L.); nmrjhkim@chungbuk.ac.kr (J.-H.K.); 6 Bio-Analytical Science, University of Science and Technology, Daejeon 34113, Korea; 7 Graduate School of Analytical Science and Technology, Chungnam National University, Daejeon 34134, Korea; 8 Research Headquarters, Korea Brain Research Institute, Daegu 41068, Korea

**Keywords:** aggregase activity, EF-Tu, EF-Ts, proteostasis, Hsp33, molecular chaperone, protein biosynthesis, trigger factor, unfoldase activity

## Abstract

**Simple Summary:**

Proteins are versatile biological macromolecules involved in most biological processes. However, because of the highly labile nature of protein structures, protein quality control (PQC) to ensure proteostasis (i.e., protein homeostasis)is difficult. Therefore, proteins of a specialized class (i.e., molecular chaperones) are required that assist in proper folding and prevent aberrant folding of other proteins. Hsp33 was originally discovered as a holding chaperone that is overexpressed upon heat shock and activated by oxidation to prevent the misfolding of client proteins. Recently, an unfoldase/aggregase activity of Hsp33 was identified in its reduced state against a specific substrate, EF-Tu, which plays a key role in protein biosynthesis in cells. The present study demonstrates that EF-Tu unfolding/aggregation by Hsp33 can be accelerated by another molecular chaperone trigger factor. Given that the unfolded/aggregated EF-Tu is finally degraded by another chaperone, Lon protease, it is likely that a chaperone network dysregulating EF-Tu operates in heat shock to attenuate protein biosynthesis, which is harmful to cell survival under stressed conditions. Therefore, the apparently contradictory chaperone function (i.e., promotion of client misfolding) of Hsp33 can also be associated with the PQC processes to ensure proteostasis in cells.

**Abstract:**

Hsp33, a prokaryotic redox-regulated holding chaperone, has been recently identified to be able to exhibit an unfoldase and aggregase activity against elongation factor Tu (EF-Tu) in its reduced state. In this study, we investigated the effect of elongation factor Ts (EF-Ts) and trigger factor (TF) on Hsp33-mediated EF-Tu unfolding and aggregation using gel filtration, light scattering, circular dichroism, and isothermal titration calorimetry. We found that EF-Tu unfolding and subsequent aggregation induced by Hsp33 were evident even in its complex state with EF-Ts, which enhanced EF-Tu stability. In addition, although TF alone had no substantial effect on the stability of EF-Tu, it markedly amplified the Hsp33-mediated EF-Tu unfolding and aggregation. Collectively, the present results constitute the first example of synergistic unfoldase/aggregase activity of molecular chaperones and suggest that the stability of EF-Tu is modulated by a sophisticated network of molecular chaperones to regulate protein biosynthesis in cells under stress conditions.

## 1. Introduction

As protein structures are labile in nature and aberrant protein folding is highly deleterious to cells, protein quality control (PQC) to ensure cellular proteostasis (i.e., protein homeostasis) is critical for cell viability [1,2,3]. Therefore, sophisticated networks of molecular chaperones, including various heat shock proteins, operate in cells as key components of the PQC machinery to achieve an optimal balance between the folding and degradation of misfolded proteins. In addition, diverse activities and functionalities of molecular chaperones are involved in almost every PQC process, including facilitation of the correct folding of proteins, prevention of protein misfolding under stressed conditions, and timely degradation of misfolded proteins and their aggregates [4,5,6,7].

The prokaryotic molecular chaperone, heat shock protein 33 (Hsp33), which was first discovered in *Escherichia coli* as a σ^32^-controlled heat shock protein [8], is prevalent in bacteria, and its eukaryotic orthologs have been identified in the kinetoplastids and green algae [9,10]. Hsp33 was originally identified as a redox-regulated molecular chaperone that requires oxidation for functional activation [11]. As the protein has a unique zinc-binding domain at its C-terminus [12], redox sensing by Hsp33 is achieved by four conserved cysteines that coordinate zinc binding [3,13,14]. Upon oxidation, the conserved cysteines in the zinc-binding redox-switch domain form disulfide bonds with concomitant release of zinc, which results in the unfolding of the C-terminal redox-switch domain and the middle linker domain [12,13,14,15,16]. Consequently, this partially-unfolded Hsp33 functions as a holding chaperone that binds to unfolding intermediates of client proteins to prevent further progression of misfolding and promote native folding [17,18,19]. In addition, dimer and high-order oligomer formation of activated Hsp33 enhances its chaperone activity [16,20,21].

In contrast to the well-defined structural mechanism for the holding-chaperone activity of oxidized Hsp33 [22], reduced Hsp33 has long been regarded as an inert state that is primed for oxidation-induced activation. However, certain specific functionalities not yet known could be expected for reduced Hsp33, considering the fact that Hsp33 is expressed at basal levels under normal conditions and the heat-induced species without oxidative stress is also the reduced form [7,11]. Furthermore, a proteomics analysis of the Hsp33 interactome suggested several promising partners, of which binding could be relevant to the reduced state of Hsp33 [23]. In this context, Bruel et al. [24] demonstrated that the overexpression of Hsp33 in an *E. coli* strain lacking the trigger factor (TF) and DnaK targeted elongation factor thermo-unstable (EF-Tu) for degradation by the protease Lon, thereby rescuing the synthetically lethal phenotype of the strain. As EF-Tu is a crucial factor for protein biosynthesis [25,26,27], the involvement of Hsp33 in EF-Tu regulation implied that the protein could contribute to PQC and cellular proteostasis [22]. Our in vitro study corroborated that Hsp33 can directly interact with native EF-Tu [28]. Notably, only the reduced form of Hsp33 was responsible for EF-Tu binding, which was critically mediated by the redox-switch domain of Hsp33 being folded. Moreover, the binding of reduced Hsp33 subsequently induced unfolding and oligomerization/aggregation of EF-Tu, which became highly susceptible to proteolysis by Lon.

EF-Tu is a translational GTPase that delivers aminoacyl-tRNA to ribosomes in cells for protein biosynthesis, and the ribosome-dissociated EF-Tu is normally stabilized by forming a complex with elongation factor thermo-stable (EF-Ts) for GDP-GTP exchange [25,26,27]. In addition, Bruel et al. suggested that another molecular chaperone, TF, might facilitate the Hsp33:EF-Tu interaction, as the cellular effect of Hsp33 on EF-Tu was more aggressive in the presence of TF [24]. Therefore, in the present study, we examined the molecular interaction of EF-Ts and TF with EF-Tu and their influence on the regulation of EF-Tu stability by Hsp33.

## 2. Materials and Methods

### 2.1. Protein Sample Preparation

The reduced zinc-bound Hsp33 and native monomeric EF-Tu were prepared as described previously [28]. We maintained Hsp33 in its reduced zinc-bound state by supplementing Zn^2+^ and reducing agents in all solutions used, whereas the oligomeric EF-Tu (^Oligo^EF-Tu) was prepared by incubating the native monomeric EF-Tu in Mg^2+^-free 50 mM HEPES buffer (pH 7.4) containing EDTA (1 mM) at 40 °C for 30 min. To generate *E. coli* EF-Ts, genomic DNA was extracted from the *E. coli* DH5α strain and used as a template for cloning. The open reading frame encoding EF-Ts was amplified via PCR using the following oligonucleotide primer pairs (*Nde*I and *Xho*I restriction sites are underlined): 5′-GGAATTCCATATGGCTGAAATTACCGCATCCCT-3′ (forward) and 5′-CCGCTCGAGTTAAGACTGCTTGGACATC-3′ (reverse). The PCR products were digested with *Nde*I and *Xho*I and then ligated into *Nde*I- and *Xho*I-restricted pCold-1 (Takara) vector plasmids. The recombinant plasmids were verified via DNA sequencing and transformed into the *E. coli* BL21(DE3)pLysS strain for protein expression of the N-terminal His_6_ tag–fused EF-Ts. To produce TF, we used *E. coli* BL21 cells transformed with pTf16 vector plasmids (Takara) that overexpress TF. The transformed cells were grown in Luria-Bertani media at 37 °C until the optical density at 600 nm reached approximately 0.7, followed by the induction of protein expression. EF-Ts expression was induced by adding 1 mM of IPTG and ZnSO_4_ at 15 °C for 24 h, whereas TF expression was induced by the addition of 0.5 g/L of *_L_*-arabinose at 37 °C for 8 h. Cells were harvested via centrifugation and disrupted via sonication. The cell lysis buffer (pH 7.4) for EF-Ts contained 50 mM Tris-HCl, 50 mM NaCl, 70 mM imidazole, 1 mM MgSO_4_, and 1 mM DTT, whereas 50 mM Tris-HCl buffer without any other additives was used for TF. After cell debris was removed via centrifugation, the recovered proteins were purified by the sequential application of the following chromatography: Ni^2+^-affinity chromatography (HisTrap FF column, Cytiva (Marlborough, MA, USA) and size-exclusion chromatography (HiLoad 16/600 Superdex 200 column, Cytiva) for EF-Ts and cation-exchange chromatography (HiTrap SP FF and HiTrap SP HP columns, Cytiva) and size-exclusion chromatography (HiLoad 16/600 Superdex 200 column, Cytiva) for TF. The concentration of the purified protein was determined using a UV spectrophotometer with a molar absorptivity at 280 nm, which was deduced from the protein amino acid sequence using the web-based tool ProtParam (https://web.expasy.org/protparam/).

### 2.2. Analytical Gel Filtration

Protein–protein interactions and oligomerization were monitored via gel filtration assay using a HiLoad 16/600 Superdex 200 column connected to an FPLC system at a flow rate of 1 mL/min. The column was pre-equilibrated at room temperature (approximately 22 °C) with a running buffer (50 mM Tris-HCl, 50 mM NaCl, 1 mM MgSO_4_, and 2 mM DTT) at pH 7.4, followed by sample loading at an injection volume of 1 mL. Prior to loading onto the column, the sample solutions containing 0.1 mM of each analyte protein were incubated at 35 °C for 30 min. The eluted proteins were detected by measuring the absorbance at 280 nm, and all individual eluents were fractionated every 5 min. Subsequently, the eluted fractions were resolved using 10% glycine-SDS-PAGE to identify the analytes.

### 2.3. Light Scattering Measurements

Protein aggregation was monitored by measuring kinetic traces of light scattering, using a Varian Cary Eclipse spectrofluorophotometer equipped with a temperature controller and a magnetic stirrer. Protein samples were dissolved in 50 mM HEPES buffer (pH 7.4) containing 150 mM NaCl, 2 mM MgSO_4_, 50 μM ZnCl_2_, and 2 mM DTT. The cuvette containing 2.23 mL of a protein (15.7 μM) sample solution was incubated in the machine at 35 °C for 2 min with continuous stirring, followed by the addition of 70 μL of another protein stock (500 μM) solution to a final concentration of 15.2 μM for each protein (i.e., equimolar titration). Light scattering was recorded for 10 min, including the initial incubation (2 min), at 400 nm with a 5-nm slit width for both excitation and emission.

### 2.4. Circular Dichroism (CD) Spectroscopy

All CD spectra were recorded on a Jasco J-710 spectropolarimeter equipped with a temperature controller, using a 0.5 mm path-length cell, with a 1 nm bandwidth and 1 s response time. For measurements of standard far-UV CD spectra, the sample solutions containing 0.2 mM of each protein in a reaction buffer (50 mM HEPES, 0.5 mM ZnSO_4_, 0.5 mM MgSO_4_, and 0.5 mM DTT) at pH 7.4 were incubated at 35 °C for 30 min, followed by dilution into a CD buffer (10 mM sodium phosphate at pH 7.4). Subsequently, the far-UV CD of the diluted sample solutions was measured at 35 °C. Three individual scans taken from 260 to 190 nm with 0.2 nm step resolution and 100 nm/min scan speed were added and averaged, followed by subtraction of the solvent CD signals. Finally, the recorded CD intensity was normalized to molar ellipticity using the following equation:

(1) $$\left[\theta \right]=\frac{10^{6}\theta }{cl}$$

where [θ] and θ are the molar ellipticity in units of deg·cm^2^·dmol^−1^ and the observed CD intensity in mdeg, respectively, with the path length l in mm at the sample concentration c in μM.

Time-course CD changes were monitored at 222 nm at every 0.5 s, with 15 μM of protein samples in 10 mM sodium phosphate buffer (pH 7.4) containing 50 μM ZnCl_2_, 1 mM MgSO_4_, 1 mM DTT, and 10 mM NaCl.

### 2.5. Isothermal Titration Calorimetry (ITC)

Binding thermodynamics were measured using a MicroCal VP-ITC instrument (Malvern Panalytical, UK) at a stirring speed of 307 rpm. All protein samples were prepared in 50 mM Tris-HCl buffer (pH 7.4) containing 50 mM NaCl, 1 mM MgSO_4_, 50 μM ZnSO_4_, and 1 mM DTT. The ITC thermogram with a single titration was monitored at 35 °C by titrating 140 μL of Hsp33 (165 μM) or equimolar (165 μM) mixture of Hsp33 and TF (Hsp33:TF) in the ITC syringe to 1.4 mL of EF-Tu (16.5 μM) in the ITC cell. The total experiment time was 20 min with a single injection at 5 min of incubation. We monitored dilution heat by a control experiment that titrated the same Hsp33 or Hsp33:TF sample to blank buffer. The recorded thermograms of the samples were processed by subtracting the corresponding heat of dilution, followed by baseline corrections. The observed peak areas were normalized by molar concentrations to yield the values of enthalpy change (Δ*H*) in kcal·mol^−1^.

Calorimetric measurements of TF (500 μM in the ITC syringe) binding to native EF-Tu and ^Oligo^EF-Tu (15 μM each in the ITC cell) were performed at 20 °C with 25 injections at a constant interval of 300 s. The reference power and initial delay were set to 10 μcal·s^−1^ and 300 s, respectively. The injection volume was 2 μL for the first injection and 5 μL for the residual injections. The dilution heat of TF was measured via titration of TF against the blank buffer. Binding isotherms were processed by baseline correction and subtraction of dilution heat, followed by fitting to a one-set of sites binding model as follows [29]:

(2) Q=n[EOligoF−Tu]tΔHV02[1+[TF]tn[EOligoF−Tu]t+Kdn[EOligoF−Tu]t                                                              −(1+[TF]tn[EOligoF−Tu]t+Kdn[EOligoF−Tu]t)2−4[TF]tn[EOligoF−Tu]t]

where *Q* is the total heat content, and Δ*H* is the enthalpy change for reactions occurring in the ITC cell. *V*_0_ and *K*_d_ denote the active cell volume and dissociation constant, respectively. [TF]_t_ and [^Oligo^EF-Tu]_t_ represent the total concentrations of TF and ^Oligo^EF-Tu, respectively, at any given time point (*t*), and *n* indicates the number of TF molecules that bind to one ^Oligo^EF-Tu molecule.

## 3. Results

**Figure 1:**
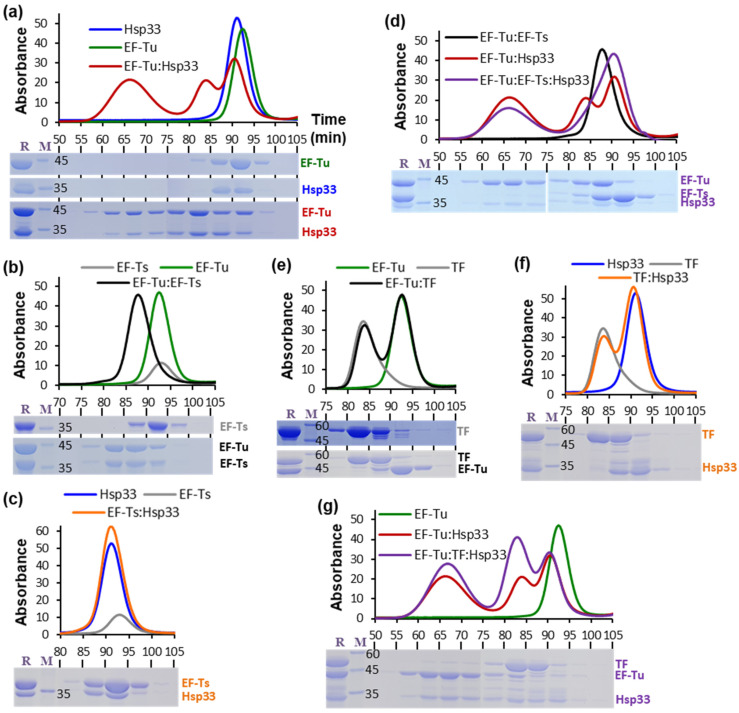
Oligomerization of EF-Tu detected via gel filtration assay. Gel-filtration (HiLoad 16/600 Superdex 200 column at 1 mL/min of flow rate) profiles are shown for EF-Tu (green in **a**,**b**,**e**,**g**), Hsp33 (blue in **a**,**c**,**f**), EF-Ts (gray in **b**,**c**), TF (gray in **e**,**f**), EF-Tu:Hsp33 (red in **a**,**d**,**g**), EF-Tu:EF-Ts (black in **b**,**d**), EF-Tu:TF (black in **e**), Hsp33:TF (orange in **f**), EF-Tu:EF-Ts:Hsp33 (purple in **d**), and EF-Tu:TF:Hsp33 (purple in **g**). Underneath the gel-filtration profiles, SDS-PAGE images are shown for individual fractions of gel filtration (lane R, mixture of the individual proteins before incubation; M, molecular size marker in kDa): EF-Tu (**a**) Hsp33 (**a**) EF-Ts (**b**) TF (**e**) EF-Tu:Hsp33 (**a**) EF-Tu:EF-Ts (**b**) EF-Tu:TF (**e**) EF-Ts:Hsp33 (**c**) TF:Hsp33 (**f**) EF-Tu:EF-Ts:Hsp33 (**d**) and EF-Tu:TF:Hsp33 (**g**). Sample solutions for the gel filtration contained 0.1 mM of each protein, which was pre-incubated at 35 °C for 30 min before loading (1 mL of injection volume) onto the column.

The molecular interactions of EF-Tu, EF-Ts, Hsp33, and TF were investigated via gel filtration assay to monitor EF-Tu oligomerization (Figure 1). Consistent with our previous observations [28], the gel filtration profile of the EF-Tu:Hsp33 mixture corroborated their complex formation and oligomerization by exhibiting three distinct elution peaks containing both proteins (red line and corresponding SDS-PAGE image in Figure 1a); the first (60–75 min fractions) and second (80–85 min fraction) peaks are accounted for by oligomeric and heterodimeric complexes of EF-Tu and Hsp33, respectively, whereas the last elution peak (90–95 min fraction) is attributable to non-complex species [28]. To examine the effect of EF-Ts on the Hsp33-mediated EF-Tu oligomerization, we confirmed that EF-Tu and EF-Ts formed a stable one-to-one complex (Figure 1b), as is generally known [27], whereas no significant interaction was observed between EF-Ts and Hsp33 (Figure 1c). Nonetheless, the oligomeric and dimeric complexes of EF-Tu and Hsp33 were evident in the gel filtration profile of the triple mixture of EF-Tu:EF-Ts:Hsp33 (purple line in Figure 1d), although the EF-Tu oligomerization (60–75 min fractions) appeared somewhat attenuated compared to that in the double mixture of EF-Tu:Hsp33 (red line). This result indicates that the oligomerization of EF-Tu occurred even in its complex state with EF-Ts via interaction with Hsp33. In addition, the monomeric EF-Ts levels (90–95 min fraction) increased compared to that in the double mixture of EF-Tu:EF-Ts (Figure 1b). Therefore, EF-Ts was considered to be dissociated from EF-Tu upon EF-Tu oligomerization by Hsp33.

Regarding TF and Hsp33, we confirmed that neither EF-Tu (Figure 1e) or Hsp33 (Figure 1f) formed a complex with TF. Nonetheless, oligomeric fractions in the gel filtration of the triple EF-Tu:TF:Hsp33 mixture contained far more EF-Tu (Figure 1g) than those in the gel filtration of the EF-Tu:Hsp33 mixture (Figure 1a). In addition, a portion of TF was present in the oligomeric fractions of EF-Tu:TF:Hsp33. Therefore, we reasoned that TF could accelerate Hsp33-mediated EF-Tu oligomerization and aggregation.

**Figure 2:**
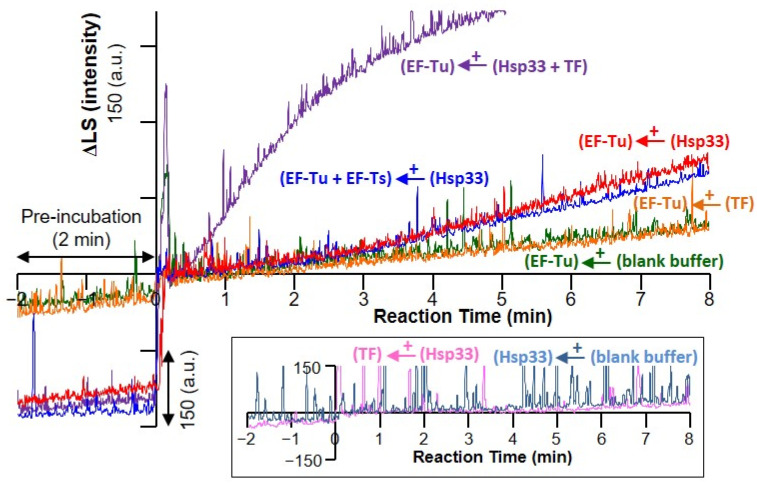
Aggregation of EF-Tu detected via light scattering. Light scattering intensity (arbitrary unit, a.u.) at 400 nm was recorded for 10 min, including the pre-incubation (2 min) and reaction monitoring (8 min) periods. Time-course change of the light scattering intensity (ΔLS) is shown for indicated protein samples. EF-Tu (green, red, purple, and orange) and EF-Tu:EF-Ts complex (blue) were incubated at 35 °C, followed by equimolar (15 μM) titration (designated as the time 0 min) of Hsp33 (red and blue), TF (orange), or both (purple). Control experiments for EF-Tu alone (green) and Hsp33 alone (light blue in the inset) were performed by adding blank buffer, whereas TF alone is presented in the pre-incubation period for subsequent addition of Hsp33 (pink in the inset).

The aggregation kinetics of EF-Tu were examined by monitoring the light scattering over time (Figure 2). Consistent with previous observations [28], the addition of Hsp33 to the EF-Tu solution caused a prompt leap, followed by a gradual increase in light scattering (red trace), which was steeper than that for the addition of blank buffer (green trace). The EF-Tu:EF-Ts complex solution also showed a comparable pattern of light scattering that increased upon the addition of Hsp33, although it showed a slightly decreased slope (blue trace). In contrast, the addition of TF had no significant effect on light scattering in the EF-Tu solution (orange line). In addition, the EF-Tu-free incubation of Hsp33, TF, and the Hsp33:TF mixture showed no significant increase in light scattering (inset in Figure 2). However, when both Hsp33 and TF were added to the EF-Tu solution, the increase in light scattering was markedly accelerated (purple trace). Similar to the gel filtration results, the light scattering results indicate that the EF-Tu oligomerization/aggregation evoked by Hsp33 is valid even in the presence of EF-Ts and is accelerated in the presence of TF.

**Figure 3:**
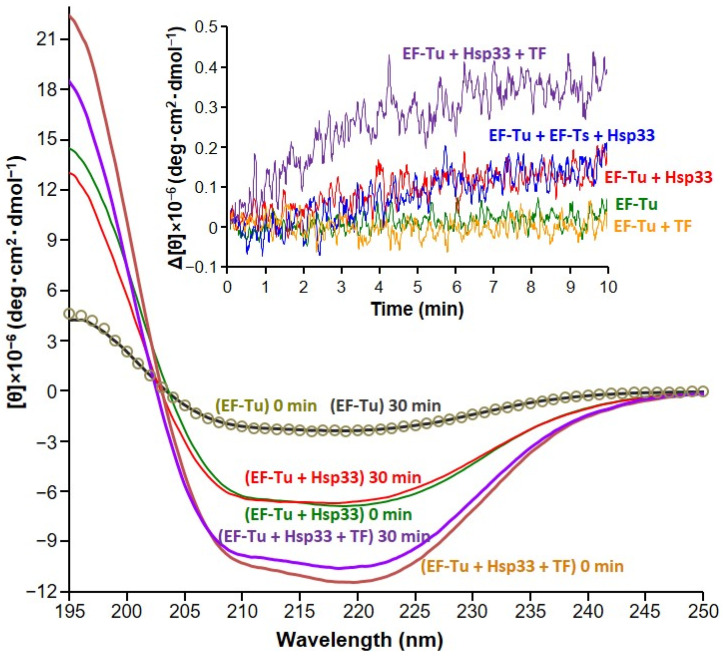
Conformational change of EF-Tu detected via CD spectroscopy. Far-UV CD spectra (main panel) were measured for the protein samples indicated in parentheses before and after 30-min incubation at 35 °C. The CD intensities measured for diluted samples (10, 5, and 3 μM for EF-Tu, EF-Tu:Hsp33, and EF-Tu:Hsp33:TF, respectively) before and after the incubation, which was performed with 0.2 mM of each protein, were normalized as molar ellipticity ([θ]). The inset shows time-dependent change of molar ellipticity (Δ[θ]) at 222 nm, for the indicated protein samples (15 μM) during incubation at 35 °C in the CD cell.

CD experiments indicated that the oligomerization/aggregation of EF-Tu was associated with its unfolding (Figure 3). Consistent with the previous observation [28], incubation of the EF-Tu:Hsp33 mixture resulted in a change in the far-UV CD spectrum (from green to red line), indicative of a partial unfolding with decreased α-helical content (e.g., decreased molar ellipticity around 222 and 195 nm), whereas EF-Tu alone showed no significant change (gray circles to gray line). It was previously proved using nuclear magnetic resonance spectroscopy that only EF-Tu was responsible for the observed unfolding in the EF-Tu:Hsp33 mixture [28]. Incubation of the triple EF-Tu:TF:Hsp33 mixture showed a more significant change in the CD spectrum (from orange to purple line). Given that Hsp33 does not interact with TF (Figure 1f), the remarkable CD change of the triple EF-Tu:TF:Hsp33 mixture could be also attributed to the unfolding of EF-Tu, implying a synergistic effect of Hsp33 and TF on the unfolding of EF-Tu. The kinetic traces of the CD change at 222 nm (inset in Figure 3) were in good agreement with those observed in the light scattering experiments (Figure 2), supporting that the aggregation of EF-Tu requires unfolding. The kinetic traces of CD change also confirmed again that the presence of EF-Ts did not inhibit Hsp33-induced EF-Tu unfolding, although it was slightly attenuated (blue vs. red trace). In addition, TF alone had no significant effect on EF-Tu unfolding (yellow vs. green trace), whereas it substantially synergized with Hsp33 for the EF-Tu unfolding (purple vs. blue trace).

**Figure 4:**
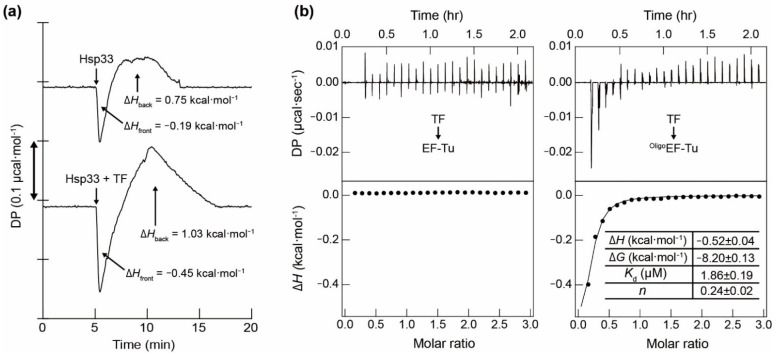
Calorimetric characterization of the TF binding to EF-Tu. (**a**) ITC thermograms obtained by a single-shot titration (added at 5 min of incubation at 33 °C) of Hsp33 (upper) and equimolar Hsp33:TF mixture (lower) to EF-Tu are shown. Differential power (i.e., heat flow) is denoted as DP. The earlier (negative) and later (positive) ITC peaks were indicated by Δ*H*_front_ and Δ*H*_back_, respectively. (**b**) ITC thermograms (upper) obtained at 20 °C by titrating TF to native EF-Tu (left) and ^Oligo^EF-Tu (right) are shown with binding isotherms (lower), where the black line indicates a fitting curve and the inset table presents the thermodynamic parameters determined for the binding of TF to ^Oligo^EF-Tu.

The synergistic effects of TF and Hsp33 were examined using ITC experiments. As observed previously, the thermogram for single titration of Hsp33 against native EF-Tu (Figure 4a) showed an unusual trace characterized by an initial negative pulse (Δ*H*_front_) and subsequent positive reaction (Δ*H*_back_), which is attributed to the exothermic binding of Hsp33 to EF-Tu, followed by an endothermic unfolding of EF-Tu [28]. The single titration of the Hsp33:TF mixture also manifested a similar thermogram pattern but caused an appreciable increase in the enthalpy change for both exothermic and endothermic reactions. To investigate the molecular mechanism of this synergistic effect, the binding of TF to EF-Tu was further monitored by a series of ITC titrations (Figure 4b). Notably, a typical binding thermogram with a micromolar *K*_d_ was observed for the TF titration to the unfolded ^Oligo^EF-Tu, whereas native monomeric EF-Tu was not responsible for TF binding (i.e., no significant reaction heat was observed). Therefore, it was inferred that TF binding to unfolded EF-Tu is associated with the cooperative regulation of the EF-Tu stability by TF and Hsp33.

## 4. Discussion

We previously demonstrated that reduced Hsp33 can directly interact with native EF-Tu, eliciting the unfolding and aggregation of EF-Tu without its own conformational change [28]. In the present study, EF-Ts, which forms a stable complex with EF-Tu, did not prevent this interaction, although the conformational stabilization of native EF-Tu by EF-Ts [27] appeared to slightly attenuate the Hsp33-induced unfolding/aggregation of EF-Tu. The interaction of EF-Tu with EF-Ts occurs through the G-domain (domain-1) and domain-3 of EF-Tu [27]. In contrast, the interaction with Hsp33 was suggested to be mediated by the G-domain/domain-2 or all three domains of EF-Tu, with the binding of the G-domain to the redox-switch domain of Hsp33 critically driving the entire molecular interaction of the two proteins [28]. Based on the present gel filtration results, as EF-Ts has stronger affinity to EF-Tu than the Hsp33:EF-Tu interaction, Hsp33 binding to the EF-Ts–complexed EF-Tu is unlikely to be achieved via competition with EF-Ts at the same binding site on EF-Tu. Our previous and present data demonstrate that reduced Hsp33 maintained binding to the unfolding/aggregating EF-Tu, whereas EF-Ts dissociated from the unfolded/aggregated EF-Tu. Collectively, we reason that Hsp33 occupies a different site, apart from the EF-Ts-binding region, on the G-domain of EF-Tu and subsequently evokes the unfolding of the G-domain, leading to the dissociation of EF-Ts.

TF in cells acts as a ribosome-associated molecular chaperone that assists the initial folding of nascent polypeptide chains emerging from the ribosome by protecting them from misfolding and aggregation as they grow [30]. TF is also abundantly free in the cytosol, where it presumably functions as a holding chaperone, like oxidized Hsp33 [30,31,32]. In the present study, as TF did not interact with either reduced Hsp33 or native EF-Tu, the observed synergistic effect of TF with Hsp33 on the unfolding/aggregation of EF-Tu could seem contradictory. However, the holding-chaperone activity usually stabilizes the partially-unfolded conformation of client proteins, and TF can bind to the partially-unfolded ^Oligo^EF-Tu. In contrast, the unfoldase action of Hsp33 against EF-Tu can be regarded as an enzyme reaction that catalyzes the equilibrium shift of EF-Tu between its folded and unfolded states [28]. Therefore, we suggest that the unfolding reaction of EF-Tu catalyzed by Hsp33 could be amplified in the presence of TF, which stabilized the unfolded state of EF-Tu.

Our previous investigation [28] showed that the ^Oligo^EF-Tu produced by Hsp33 binding was readily recognized and digested by the protease Lon, which also belongs to the heat shock regulon of bacteria [33]. Therefore, from a biological perspective, the present results provide a plausible scenario for the interplay between molecular chaperones to regulate protein biosynthesis for cell survival under heat stress. Since the aberrant folding and cytotoxic aggregation of proteins are promoted by heat stress, repression of protein synthesis by elongation pausing is a common cellular response to heat shock [34]. As Hsp33 binds to EF-Tu, both free and EF-Ts–complexed EF-Tu would lose their functionality for translation elongation because of aberrant conformational change. The chaperone function of TF for protein biosynthesis could also be involved in this elongation posing process by promoting Hsp33-induced EF-Tu unfolding/aggregation. Subsequently, the EF-Tu-specific degradation by Lon would enable Hsp33 and TF to be recycled to further attenuate translation elongation. This possible in vivo regulation of EF-Tu by Hsp33 and TF might be linked to other molecular systems, particularly those including the foldase chaperone DnaK. The functional cooperation between TF and DnaK is essential for the folding of nascent polypeptides and the refolding of the folding-competent intermediate [30,31,32]. Crosstalk with DnaK is also required for the redox-regulated holding function of Hsp33 to turn its bound substrates (i.e., the oxidation-induced unfolding intermediates) over to DnaK when oxidized Hsp33 reverts to its reduced form upon subsided oxidative stress [19]. However, reversed crosstalk would operate for the proposed action of reduced Hsp33 in heat shock, since EF-Tu is a likely client of DnaK under normal conditions; for example, the eukaryotic counterpart of DnaK (Hsp70) interacts with eukaryotic EF-Tu (EEF1A1) under normal conditions and acts as a central repressor of elongation pausing [34]. Given that the interaction between the cognate pair of EF-Tu-DnaK (EEF1A1-Hsp70) in eukaryotes was confirmed to be significantly weakened during heat stress [34], heat shock could serve as an effective signal for EF-Tu to switch its binding chaperone from DnaK to Hsp33. Overall, although the unfoldase/aggregase activity of reduced Hsp33 is unusual in general molecular chaperones [28], its collaboration with other chaperones could contribute to cellular proteostasis in heat shock by downregulating protein biosynthesis via dysregulation of EF-Tu.

EF-Tu in a photosynthetic cyanobacterium has been identified as an oxidation target of cellular reactive oxygen species, resulting in oligomerization/aggregation [35]. Considering that Hsp33 unfolds upon oxidation to exhibit its holding-chaperone activity, it might be possible that the function of Hsp33 switches under oxidative stress to prevent EF-Tu from aggregation, which can address the observation that *E. coli* Hsp33 did protect against EF-Tu degradation in *Vibrio cholerae* under oxidative conditions [36]. However, Lon is unlikely to be involved in this currently uncharacterized system as our previous study showed that oxidized Hsp33 could also be an efficient substrate of Lon [28]. Alternatively, the chaperone network, including Hsp33, TF, and Lon, for the PQC processes might be differently modulated depending on the organism and/or the type of cellular stress; however, this remains to be investigated. In addition, given the base-level expression of Hsp33 under non-stressed conditions [7], reduced Hsp33–mediated specific functionality might also be engaged as a component of the cellular PQC machinery under normal conditions. However, it remains to be verified whether EF-Tu regulation by Hsp33 is compatible with such housekeeping roles.

## 5. Conclusions

EF-Tu and its cognate partner EF-Ts are key factors in the ribosomal biosynthesis of proteins in cells. We have previously demonstrated in vitro that Hsp33 in its chaperone-inactive, reduced state binds to and subsequently evokes the unfolding and aggregation of EF-Tu, which in turn becomes susceptible to proteolytic degradation by Lon [28]. Following the previous investigation, the present study elucidated that the Hsp33-induced unfolding/aggregation is also valid for the stabilized EF-Tu in complex with EF-Ts. Furthermore, TF was observed to amplify the Hsp33-induced unfolding/aggregation of EF-Tu, probably via its binding to the partially-unfolded EF-Tu. Therefore, we conclude that the collaborative action of molecular chaperones, including Hsp33, TF, and Lon, would be feasible in cells for the dysregulation of the EF-Tu functionality, which can contribute to combating heat stress via global downregulation of protein biosynthesis.

## Data Availability

Not applicable.

## Abbreviations

CD	circular dichroism
DTT	1,4-dithiothreitol
EDTA	ethylenediaminetetraacetic acid
EF-Ts	elongation factor thermo-stable
EF-Tu	elongation factor thermo-unstable
FPLC	fast protein liquid chromatograpy
HEPES	2-[4-(2-hydroxyethyl)piperazin-1-yl]ethanesulfonic acid
Hsp33	heat shock protein 33
IPTG	Isopropyl β-D-1-thiogalactopyranoside
ITC	isothermal titration calorimetry
PCR	polymerase chain reaction
PQC	protein quality control
SDS-PAGE	sodium dodecyl sulfate-polyacrylamide gel electrophoresis
TF	trigger factor
Tris-HCl	tris(hydroxymethyl)aminomethane hydrochloride
